# Age-Related Changes in the Regenerative Potential of Adipose-Derived Stem Cells Isolated from the Prominent Fat Pads in Human Lower Eyelids

**DOI:** 10.1371/journal.pone.0166590

**Published:** 2016-11-17

**Authors:** Xinhai Ye, Caihe Liao, Guangpeng Liu, Yipin Xu, Jian Tan, Zhenshun Song

**Affiliations:** 1 Department of Plastic and Reconstructive Surgery, Shanghai Tenth People's Hospital, Tongji University School of Medicine, Shanghai, China; 2 Department of Hepatobiliary and Pancreatic Surgery, Shanghai Tenth People's Hospital, Tongji University School of Medicine, Shanghai, China; Instituto Butantan, BRAZIL

## Abstract

The existence of multipotent adipose-derived stem cells isolated from human orbital fat (OF) tissue has shown great therapeutic potential in tissue engineering and regenerative medicine. But the use of stem cells for therapeutic applications is influenced by their proliferative and differentiation potentials, which may be affected by the age of the donor. So far there is little knowledge about the effects of donor age on the biological properties of human orbital adipose-derived stem cells (OASCs). The intraorbital fat protrusion in the lower eyelids occurs as an aging process, and the protruded fat is routinely removed during aesthetic surgeries. Based on the ease of OF harvest and the availability of OASCs, we investigated in this study the relationship between age and the differentiation and proliferation potentials of human OASCs. Human orbital adipose samples were harvested from young (with normal lower eyelid appearance) and old donors (having protruded fat pads in the lower eyelids). The morphological properties of orbital adipocytes were assessed and the fat cell size displayed a decreasing trend with advancing age. OASCs were isolated from the fat samples, expanded *in vitro* and cultured under appropriate inducive conditions. Compared to the young cells, although no difference was found in the cell yield and phenotype expression, aged OASCs showed fewer progenitor cell numbers, reduced proliferative rates, increased senescent features and decreased differentiation potentials towards adipogenic, osteogenic and chondrogenic lineages. Our data suggested that using autologous OASCs from elderly patients for potential therapeutic purposes might be restricted.

## Introduction

Adipose-derived stem cells (ASCs) are a population of postnatal progenitor cells existing abundantly in adipose tissue, which can expand rapidly *in vitro*, possess multilineage differentiation potentials and remain stable over long-term culture [1,2]. Compared to the adult stem cells derived from other tissue sources, such as bone marrow, muscle, ligament, synovium and dental pulp, the advantages of ASCs include the large quantities of fat tissue, minimal patient discomfort, little donor-site morbidity and ease of cell isolation [3,4]. Thus, there has been growing interest in applying ASCs as a potential cell source in the field of tissue engineering and regenerative medicine during the past two decades.

The human body can be segmented into various depots of fatty tissue based on the anatomic region [5]. These depots vary in the amount of adipose tissue and may produce ASCs with different biological characteristics. The orbital cavity contains a highly specialized adipose depot which differs from the subcutaneous fat (SF) developmentally and functionally [6]. The existence of human orbital adipose-derived stem cells (OASCs) also has been proven. In addition to differentiating into adipocytes, chondrocytes and osteoblasts, these cells also can differentiate towards the corneal epithelial, smooth muscle and neuronal lineages under specific inductive conditions, showing great therapeutic potential in treating the orbital and ocular diseases [6–9].

The intraorbital fat prominence of the lower eyelids occurs as an aging process, and has been a frequent complaint prompting old people to seek cosmetic surgery called blepharoplasty [10,11]. So it becomes an interesting issue to investigate changes in the characteristics of OASCs isolated from the prominent orbital fat (OF) pads. Identifying this issue is of significance when promising OASC-based therapies are designed for elderly patients. Utilization of stem cells for potential therapeutics is influenced by their proliferative and differentiation capacities, which may be dramatically affected by the age of patient or donor. To date, however, little literature has investigated the relationship between age and the biological properties of OASCs.

In this study, we tested whether the proliferative and differentiation potentials of human OASCs were affected by donor age. First, the OF samples were harvested from young and old patients with the baggy lower eyelids during routine blepharoplasty. The histological evaluation was performed to observe the morphological changes of fat cells in relation to age. Next, OASCs were isolated from OF samples and expanded *in vitro*. The cell yield, phenotype expression, growth kinetics and senescent features were assessed respectively. Finally OASCs were cultured under adipogenic, chondrogenic and osteogenic conditions to compare their differentiation potentials towards multilineage cell fates.

## Materials and Methods

### Ethics statement

The protocol for this study was approved by the Research Ethics Committee of Tongji University School of Medicine (No. 2015–0083) and conformed to the principles outlined in the Declaration of Helsinki. All participants have provided their written informed consent (as outlined in PLOS consent form) to participate in this study and to publish their case details.

### Harvest of OF samples

This study was conducted from May in 2015 to April in 2016. Adipose tissue samples were surgically harvested from the central compartments of OF pads, which are usually discarded after removal, during the lower eyelid blepharoplasty in 20 female healthy patients who had given their written informed consent. Patients with obesity (BMI > 25 kg/m^2^), orbital disease or endocrine disease were excluded from this study. The donors were divided into two age groups: Group A (age range, 20–38 and mean age, 26.33 ± 5.48 years old, n = 10) and Group B (age range, 50–67 and mean age, 56.44 ± 5.83 years old, n = 10). Patients in Group A had normal lower eyelid appearance while those in Group B had obvious OF protrusion in the lower eyelids. Fat samples were preserved on ice under aseptic condition, transported to the laboratory immediately after surgery and processed within 6 hours.

### Evaluation of adipocyte morphology

After weight measurement, four specimens in each group were fixed in 10% formalin overnight, embedded in paraffin, sectioned in 5-μm thickness and processed for routine hematoxylin and eosin (HE) staining. Images were taken in triplicate for each specimen using an optical microscope (IX70, Olympus, Tokyo, Japan). The photographs were processed with the green channel to enhance edge prominence using the Image-Pro Plus software (v6.0, Media Cybernetics, Silver Spring, MD, USA). Three main parameters were measured to evaluate the adipocyte morphology as previously described [12]: cell diameter (length of the longest line joining two points and passing through the centroid), perimeter and area. Approximate 300 adipocytes were measured for each histological image.

### Isolation and expansion of OASCs

OASCs were isolated and expanded as previously reported with minor modification [6]. Briefly, after washed in phosphate buffer solution (PBS, pH 7.4, Sigma, Shanghai, China) extensively, another six OF samples from each group were minced with sterile scissors and digested with 0.1% type I collagenase solution (Worthington Biochemical Corp, Lakewood, NJ, USA) at 37°C for 60 min under constant shaking. The upper layer of adipocytes was removed by aspiration and the remaining cells were filtered through a 100-μm and then a 40-μm nylon strainer (BD Bioscience, Franklin Lakes, NJ, USA). Filtered cells were centrifuged for 5 min at 400 *g*, resuspended in 3 mL of the growth medium (GM, containing low-glucose Dulbecco's modified Eagle's medium (LG-DMEM, Gibco, Grand Island, NY, USA) and 10% fetal bovine serum (HyClone, Logan, UT, USA) plus 1% antibiotic/antimycotic). The viability of cells was assessed using the trypan blue exclusion method (Sigma) and the nucleated cell yield was derived by dividing total viable cells by the weight of adipose tissue digested (in gram). For ASC expansion, single-cell suspension was plated into 35-mm culture dishes (Falcon, B&D Bioscience, San Jose, CA, USA) at a density of 50,000 cells/cm^2^. The GM was replaced 48 hours after cell plating to remove the non-adherent cells (red blood cells and fat cells) and changed twice a week thereafter. Cells adherent on the plastic substrate were designated as OASCs of passage 0 (P0). Upon reaching approximate 80–90% confluence, cells were detached with 0.05% trypsin/0.5 mM EDTA (Sigma), replated into 6-well culture plates (Falcon) at a density of 5,000 cells/cm^2^, and subcultured as the first-passaged cells (P1).

### Cell colony forming and growth kinetics

Colony forming unit-fibroblast (CFU-f) assay was performed using methods described previously [13]. Briefly, nucleated cells freshly isolated from OF tissue were plated into 6-well culture plates (Falcon) at a density of 1,000 cells/cm^2^ and cultured in the GM, which was changed twice a week. On the 14^th^ day after plating, the total number of cell colonies (CFU-f, a cluster of at least 50 adhered and fibroblast-like cells) was counted using Crystal Violet staining (Sigma).

To assess the proliferative potential of ASCs, cells from P1 were seeded into 6-well plates at a density of 5,000 cells/cm^2^, subcultured in GM and passaged similarly for a total of 9 passages. The cell population doubling time was determined using the following formula: (days in culture)/[log_10_(NH)-log_10_(NI)]/log_10_(2), where NI is the inoculum cell number and NH is the cell harvest number. Once the cells were unable to reach confluence or a doubling time of over 100 hours was obtained in two consecutive passages before achieving the 9^th^ passage, the culture was considered to have failed at that passage [14].

### Flow cytometry of OASCs

The phenotypic characterization of OASCs was performed using a FACScan cytometer (Coulter Epics Altra, Becton Dickson, San Jose, CA, USA). Briefly, cells of passage 2 (P2) were trypsinized and resuspended in the flow cytometry buffer (PBS containing 0.1% FBS and 0.02% natrium azide). After blocked with human immunoglobulin, cell aliquots (1×10^5^) were incubated with the following fluorescein isothiocyanate (FITC)-conjugated or phycoerythrin (PE)-conjugated monoclonal antibodies: CD14-FITC, CD19-FITC, CD34-FITC, CD45-PE, CD73-PE, CD90-FITC and CD105-FITC. Labeled cells were analyzed by flow cytometry and non-specific IgG stained cells were used as isotype controls (all antibodies from Santa Cruz Biotechnology, Dallas, TX, USA).

### Cellular senescence of OASCs

The mRNA level of genes related to cell aging, p21 and p53, were quantified by real-time PCR assay [15]. Briefly, total RNA was extracted from OASCs of passage 3 using the RNeasy Mini Kit (Qiagen, Valencia, CA, USA). RNA samples were reverse-transcribed to cDNA using Oligo dT primers and the final cDNA was subjected to real time PCR (7300 Real-Time PCR System, Applied Biosystems, Foster City, CA, USA). Fold changes in gene expression level were calculated by the 2^-ΔΔCt^ method and the results were normalized to the expression of an internal control, β-actin. The PCR primer sequences used in this study were listed in Table 1, with primer specificity confirmed on the NCBI Primer-BLAST website (www.ncbi.nlm.nih.gov/tools/primer-blast).

**Table 1:** Primer sequences and conditions for real time PCR.

Gene	Primers (F = forward; R = reverse)	Amplicon size (bp)	Annealing temperature (°C)
p21	F: 5’-GCGATGGAACTTCGACTTTGT -3’	314	58
	R: 5’-GGTAGAAATCTGTCATGCTGGTC -3’		
p53	F: 5’-GCTTTGAGGTGCGTGTTTGTG -3’	125	60
	R: 5’-GTTGGGCAGTGCTCGCTTAG -3’		
PPAR-γ2	F: 5’- CCTCGGTGACTTATCCTGTGGT -3’	265	60
	R: 5’- GACATCCCGACAGAAAGGCAC -3’		
leptin	F: 5’- GGACTTCATTCCTGGGCTCC -3’	134	60
	R: 5’- GGAGGTTCTCCAGGTCGTTG -3’		
LPL	F: 5’-ATGGCTGGACGGTAACAGGAAT-3’	106	58
	R: 5’-GACAGCCAGTCCACCACAATGA-3’		
aggrrecan	F: 5’-AACTGCGGTGGCAACCTCCTG-3’	293	60
	R: 5’-TCCCGGGCGGTAGTGGAATAC-3’		
Collagen type II	F: 5’-GACAATCTGGCTCCCAAC-3’	257	55
	R: 5’-ACAGTCTTGCCCCACTTAC-3’		
β-actin	F:5’-CACCCAGCACAATGAAGATCAAGAT -3’	317	60
	R:5’- CCAGTTTTTAAATCCTGAGTCAAGC -3’		

PPARγ2, peroxisome proliferator-activated receptor γ2; LPL, lipoprotein lipase.

Moreover, the senescence-associated β-galactosidase (SA-β-gal, Sigma) staining was performed to indicate replicative senescence in OASCs [16]. The ratio of SA-β-gal-positive cells was calculated as (Np/Nt)×100%, where Np is the number of positive-staining cells and Nt is the total cells counted within the image. At least five different microscopic images were randomly taken for each cell sample.

### Adipogenic differentiation of OASCs

When OASCs of passage 3 (P3) from both young and old donors reached nearly confluence, adipogenic differentiation was induced by replacing GM with the adipogenic medium (AM, consisting of GM plus 0.5mM isobutyl-methylxanthine, 10 mM insulin, and 200 mM indomethacin, all from Sigma). AM was changed twice a week and the intracellular lipid accumulation was assessed by oil red O staining (Sigma) after 14-day differentiation. For quantification measurement, the number of oil red-positive-staining cells was displayed as the percentage of the total cells counted within the image. Cells cultured in GM for 2 weeks were served as controls.

Furthermore, the mRNA level of adipogenesis-related genes, peroxisome proliferator-activated receptor γ2 (PPARγ2), leptin and lipoprotein lipase (LPL), was quantified by real-time PCR, with the primer sequences listed in Table 1.

### Osteogenic differentiation of OASCs

When OASCs of P3 grew nearly confluency, osteogenic differentiation was initiated by cultivating the cells in the osteogenic medium (OS, including GM supplemented with 0.1 μM dexamethasone, 50 μM ascorbate-2-phosphate, and 10 mM β-glycerophosphate (all from Sigma)). The induction culture was maintained for 2 weeks with medium changed every 3 days, whereas cells cultured in GM were served as control. Alizarin red staining and von Kossa staining were then performed to observe the calcification of extracellular matrix (ECM) [17,18]. For quantitative measurement, the percentage of area stained positively by Alizarin red was determined using Image-Pro Plus software by quantifying the area stained and the actual area in each view respectively [18]. Then the stain was solubilized with 10% cetylpyridinium chloride monohydrate for 30 min at room temperature. Supernatants were transferred to 96-well plates (Falcon) and the Alizarin red uptake was measured for absorbance at 562 nm using an ELISA plate reader (Varioskan, Thermo Electron, Waltham, MA, USA) as previously described [19].

### Chondrogenic differentiation of OASCs

OASCs of P3 were trypsinized, counted, and centrifuged into cell pellets (15×10^6^ per pellet) in 15 ml conical tubes (Falcon). After culture in GM for 24 hours, chondrogenesis was induced using the chondrogenic medium (CM, containing high glucose DMEM supplemented with 10% FBS, 10 ng/mL transforming growth factor-β1 (TGF-β1), 100 ng/ml insulin-like growth factor (IGF), 40 ng/mL dexamethasone and 6.25 mg/ml transferrin (all from Sigma except FBS from HyClone)). Cells were cultured in CM or GM for 3 weeks and half media were changed twice a week. Afterwards, the pellets were fixed with methanol, embedded in paraffin and processed for Alcian blue staining. After visualization, the blue dye was extracted with 6 M guanidine HCl and its absorbance was read at 620 nm [20]. Real time PCR assay was also performed to detect the expression of cartilage specific genes, collagen type II and aggrecan, whose primers sequences are listed in Table 1.

### Statistical analysis

All data collected were presented as mean ± standard deviation (SD). One-way analysis of variance and the Student-Newman-Keuls test were used to determine possible significant differences (*p* < 0.05) between groups.

## Results

### Morphological observation of orbital adipocytes

**Fig 1:**
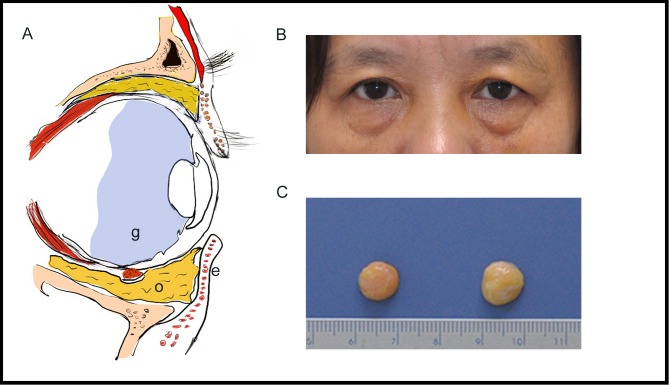
OF anatomy and observation. (A) Sagittal section of the human orbit showing OF location in the lower eyelid (e: lower eyelid skin and orbicularis oculi muscle; o: orbital fat; g: globe). (B) Photograph of a 56-year-old woman having baggy lower eyelids (who has given written informed consent to publish her picture in this manuscript). (C) Gross observation of OF tissue removed in lower eyelid blepharoplasty. The fat from young donor (right) appeared more yellow in color than that from the aged donor (left) (scale bar: 1mm).

**Fig 2:**
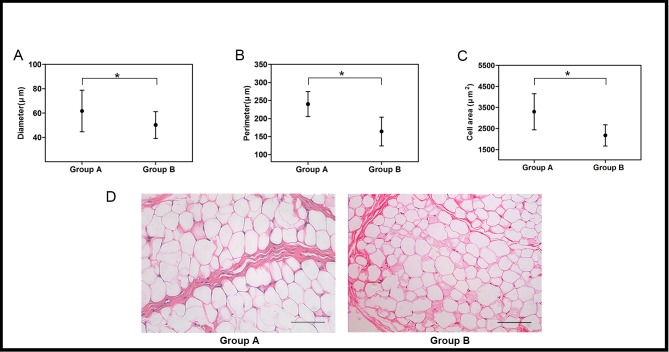
Orbital adipocyte morphology. The mean diameter (A), perimeter (B) and area (C) of fat cells from young donors (Group A) were higher than those from old donors (Group B). Data were expressed as mean ± SD with n = 4 (*: *p* < 0.05). (D) Representative HE staining images of OF samples. The adipocyte arrangement and fibrous structure in Group B were looser than those in Group A (scale bar: 100μm).

Normally OF depots in the lower eyelids are situated behind the orbicularis muscle, serving as a barrier between the eyeball and the eyelid. At the middle or early old age, it tends to migrates anteriorly to cause a baggy appearance of the lower eyelids. By gross observation, OF tissue taken from the lower lids in Group A was more yellow in color than that in Group B (Fig 1A–1C). The average mass of fat samples was (0.412 ± 0.189) g and (0.451 ± 0.211) g for the two groups, respectively. No significant difference was observed regarding the tissue mass between groups (*p* > 0.05). Histologically, it was found that the septum tissue separating the adipose lobules was dense in the young group and became loose in the old group. Compared to Group A, adipocytes within the fatty lobules in Group B were not arranged tightly and their sizes were more irregular (Fig 2D). The mean diameter, perimeter and area were identified to depict the adipocyte morphology. All the parameters evidenced significantly decreasing trends with advancing ages (*p* < 0.05, Fig 2A–2C).

### Cell yield and growth kinetics

**Fig 3:**
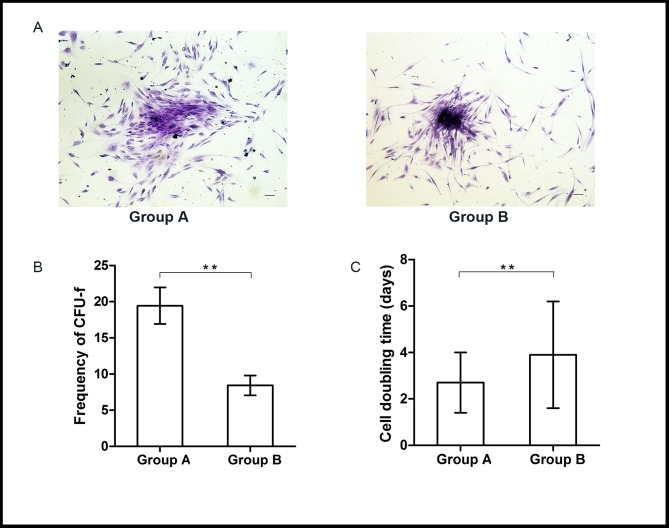
Growth characteristics of OASCs. The CFU-f numbers of primary OASCs were determined at day 14 after plating using Crystal Violet staining method. (A) OASCs in Group A formed bigger colonies containing more cells than those in Group B (scale bar: 100μm). Both the CFU-f numbers in the primary culture (B) and the cell doubling time in subculture (C) showed significant differences between two groups. Data were presented as mean ± SD with n = 6 (**: *p* < 0.01).

The yield of viable nucleated cells isolated from OF tissue in Group B was lower than that in Group A ((0.68 ± 0.13) *vs*. (0.75 ± 0.17) ×10^6^ cells/g), but the difference was not statistically significant (*p* = 0.205). In Group A, the cell colonies could be detected apparently as early as 48 hours after primary plating and these cells proliferated rapidly. In contrast, colonies usually formed 120 hours or more after seeding in Group B (Fig 3A) and OASCs grew relatively slowly. When the CFU-f numbers were counted on the 14^th^ day in culture, more and bigger colonies could be observed in Group A than in Group B (19.45 ± 2.52 *vs*. 8.42 ± 1.37, *p* < 0.01) (Fig 3B). Passaged cells from both groups exhibited similar homogeneous fibroblast-like morphology, but the donor age was found to have a significantly adverse effect on the cell proliferative rate. Totally four of 6 cell lines in Group B ceased to proliferate at the 6^th^ passage, and only two reached the 7^th^ passage. In Group A, 5 cell lines from the 6 donors reached the 9^th^ passage and one failed at the 7^th^ passage. When compared in pairs from P1 to P6, approximate 12–14 cell doublings were achieved in each group. But the average doubling time of OASCs in Group A was obviously shorter than that of Group B (2.7 ± 1.3 days *vs*. 3.9 ± 2.3 days, *p* < 0.01) (Fig 3C).

### Flow cytometry analysis

The cell phenotype profile of OASCs from young and old donors was compared using flow cytometry analysis. Our results showed that cells in both groups were negative for the hematopoietic, endothelial or lymphocyte markers (CD14, CD19, CD34 and CD45), and positive for the adult mesenchymal stem cell (MSC) markers (CD73, CD90 and CD105). No significant difference in the MSC surface antigen expression was observed between groups (*p* > 0.05, data not shown).

### Cellular senescence

**Fig 4:**
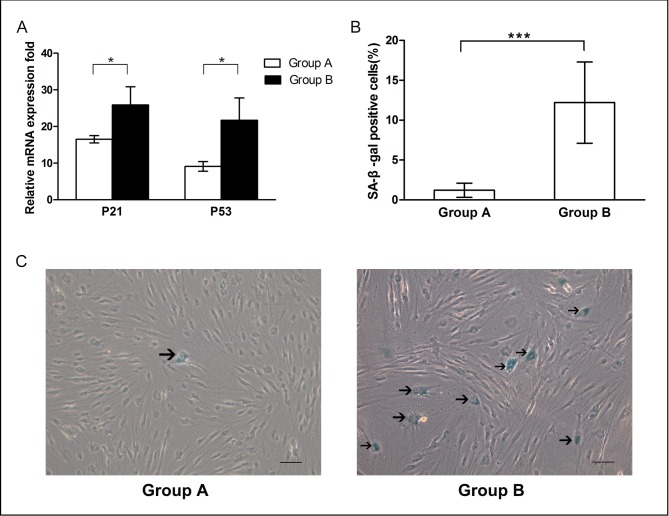
Senescence features of OASCs. (A) The expression levels of p21 and p53 mRNA were higher in Group B than those in Group A. (B) Percentage of SA-β-gal positive staining cells calculated in two groups revealed that more cells underwent replicative senescence in Group B. Results were expressed as mean ± SD with n = 6 (*: *p* < 0.05, **: *p* < 0.01). (C) SA-β-gal staining images of OASCs. Arrows indicate the positive staining cells (scale bar: 100μm).

The mRNA levels of p21 and p53 genes and the activity of SA-β-gal were measured as biomarkers to evaluate OASCs' senescence. As shown in Fig 4A, the mRNA expression of both senescence-related genes was significantly higher in Group B than that in Group A (*p* < 0.05). In agreement with the real time PCR results, increased SA-β-gal accumulation was visible in the cells isolated from the old donors with prominent lower eyelids. The ratio of SA-β-gal positive cells in Group A was only one-tenth of that in Group B, suggesting that more cells underwent replicative senescence with aging (1.2% ± 0.9% *vs*. 12.2% ± 5.1%, *p* < 0.01) (Fig 4B and 4C).

### Adipogenic differentiation potential

**Fig 5:**
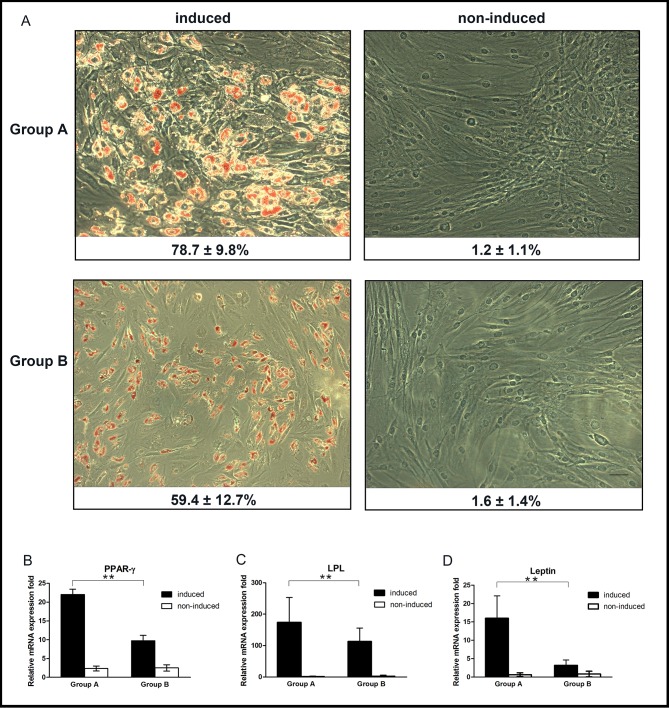
Adipogenic differentiation of OASCs. (A) Oil red O staining was performed after culturing OASCs in adipogenic media for 14 days. The ratio of positive staining cells in each group is indicated below its corresponding image (scale bar: 100μm). Real time PCR analysis confirmed the adipogenic differentiation of OASCs in contrast with the non-induced cells. Compared to the young donor cells, the expression levels of PPARγ2 (B), LPL (C) and leptin (D) mRNA were all significantly reduced in the aged cells. Data were expressed as mean ± SD with n = 6 (*: *p* < 0.05, **: *p* < 0.01).

Adipogenic differentiation of OASCs was confirmed by the oil red O staining after 14-day induction. Compared to the non-induced cells, which exhibited long spindle-like morphology and failed to accumulate any lipid, differentiating OASCs assumed an expanded morphology consistent with adipocytes and contained red staining lipid droplets within the cytoplasm (Fig 5A). Quantitative analyses showed that, after adipogenic induction, the percentage of oil red O-positive staining cells in Group A was obviously higher than that in Group B (78.7% ± 9.8% *vs*. 59.4% ± 12.7%, *p* < 0.05). The real-time PCR results also revealed significantly lower expression levels of PPARγ2, leptin and LPL in the aged group than those in the young group (*p* < 0.01, Fig 5B–5D).

### Osteogenic differentiation of OASCs

**Fig 6:**
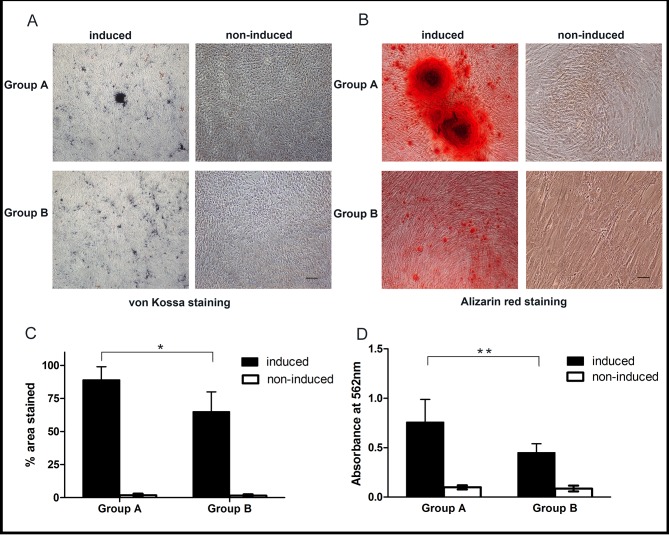
Osteogenic differentiation of OASCs. Positive von Kossa staining (A) and Alizarin red staining (B) demonstrated the calcified nodules (black and red, respectively) in the induced cells of both groups compared to the non-induced cells (scale bar: 100μm). The percentage of positively stained area with Alizarin red (C) and calcium content in cells measured by the light absorbance of Alizarin red stain (D) evidenced significant age-related decreases in osteogenic capacity of OASCs. Data were expressed as mean ± SD with n = 6 (*: *p* < 0.05, **: *p* < 0.01).

Osteogenic potential was determined by von Kossa staining and Alizarin red staining to indicate mineralization of ECM. Both measures demonstrated positive staining in osteo-induced cells in contrast to the non-induced cells, indicating the osteogenic differentiation of OASCs from both young and old donors (Fig 6A and 6B). By quantifying the percent area stained with Alizarin red, the induced young OASCs displayed (86.7 ± 10.0)% area positively stained, higher than the (65.1 ± 15.1)% area stained in OASCs from the elderly donors (*p* < 0.05, Fig 6C). The calcium deposition in cells was semi-quantitatively measured using a colorimetric assay of the solubilized Alizarin red stain, and the results also showed a negative effect of donor age on the osteogenic potential of OASCs (0.78 ± 0.23 *vs*. 0.45 ± 0.09 OD units for the induced cell comparison, *p* < 0.01) (Fig 6D).

### Chondrogenic differentiation of OASCs

**Fig 7:**
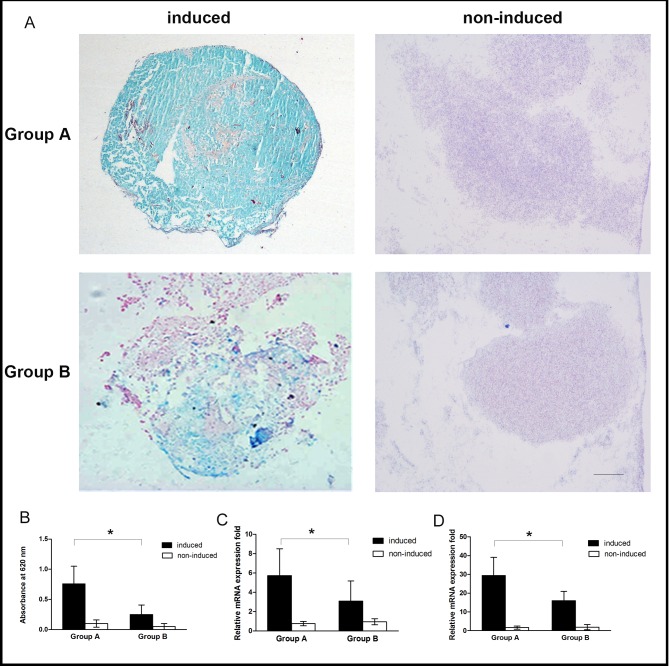
Chondrogenic differentiation of OASC pellets. (A) Alcian blue staining was determined after chondrogenic induction for 3weeks. Stronger positive staining (blue) found in Group A than in Group B (scale bar: 200μm). Quantitatively, the light absorbance of Alcian blue uptake (B) and mRNA expression levels of cartilage-related genes of collagen type II (C) and aggrecan (D) were all significantly higher for the induced cells in Group A than those in Group B. Data were expressed as mean ± SD with n = 6 (*: *p* < 0.05).

OASC pellets were chondrogenically cultured for 3 weeks in CM and sections of the pellets were stained with Alcian blue. As illustrated in Fig 7A, positive Alcian blue staining confirmed the chondrogenic differentiation of OASCs compared to the non-induced cells, with fewer positively-stained areas found in the aged cell pellets. When colorimetrically quantified, Group A demonstrated significantly more uptake of Alcian blue staining per induced cell pellet than Group B (0.76 ± 0.29 *vs*. 0.25 ± 0.16 OD units, *p* < 0.05) (Fig 7B). The real time PCR results also showed higher mRNA expression of aggrecan and collagen type II genes in Group A than that in Group B, supporting that donor age also influenced adversely on the chondrogenic potential of OASCs (*p* < 0.05, Fig 7C and 7D).

## Discussion

The multi-lineage differentiation capacity of OASCs has shown great therapeutic potential in the field of ophthalmology and regenerative medicine [6–9]. Because such promising applications involve the treatment of diseases and conditions found in an aging population, hindered by stem cell senescence and dysfunction in the natural aging process, the donor or patient age must be accounted for before the autologous stem-cell-based therapeutics entering the clinic. In this study, we evaluated the biological properties of OASCs isolated from the prominent OF pads in lower eyelids of elderly people, which can be obtained conveniently in routine blepharoplasty. To the best of our knowledge, this is the first report to characterize the age-related effects on the regenerative capabilities of OASCs.

Little consensus is achieved in literature regarding the impact of donor age on the characteristics of ASCs isolated from the subcutaneous fat (SF) tissue. Some studies indicated that aging has less effect on the self-renewal and differentiation potentials of ASCs [13,15,17,21–24], while others reported reduced viability, proliferation and differentiation in aged ASCs compared to young cells [16,20,25,26]. Multiple factors may contribute to the discrepant conclusions between these studies, such as the sample size, donor gender and age, as well as different experimental parameters and conditions.

On the other hand, OF is quite distinct from SF though they are both white fat tissue and have similar lobule structures [27]. Developmentally, SF is mesodermally derived and OF is developed from the combination of mesodermal and ectodermal cells [28]. Functionally, OF is not sensitive to diet or exercise. The main role of OF is to provide mechanical protection and preserve movement freedom for the ocular globe and intra-orbital structures, and its role as energy reserve to regulate metabolic activity is secondary [12,27]. Both SF and OF experience dramatical changes with aging. SF tends to decline substantially in advanced old age, but OF often protrudes forwards into the lower eyelids after middle age and undergoes little diminution at old age [29,30]. By far, however, there has existed little understanding as to the characteristic changes of OASCs derived from the prominent OF pads of the elderly people.

At the histological level, we found that the fibrous septum surrounding OF lobules became looser and the adipocytes appeared smaller in the old-donor group than those in the young group (Fig 2D). The rarefraction of septum structure correlated with age is not unpredicted, which is a contributing factor to the OF herniation in the lower eyelids [31]. And the fat cell size turning irregular and smaller may indicate the reduced capacity to accumulate lipid and reserve energy [29,30]. This was confirmed by the macroscopic appearance of OF. Samples from young donors were more yellow in color than those from the old group (Fig 1C).

After morphological assessment, OASCs were isolated and expanded *in vitro*. Cells from both groups exhibited a fibroblastic morphology and expressed a similar phenotype profile specific for MSC markers (CD73, CD90 and CD105). The findings were consistent with that reported for ASCs from SF depots [4,13], indicating that OASCs' morphology and phenotype were independent of donor age.

Although the numbers of viable nucleated cells freshly isolated from OF samples were comparable between two groups, we found that the progenitor cell frequencies (the ability to form colonies) declined with age based on the CFU-F results. OASCs from young donors could produce more colonies containing larger numbers of cells (Fig 3A). When the cell growth kinetics were compared in pairs from passage 1 to passage 6, it was found that the proliferation rate of young OASCs was significantly greater than of the aged cells (Fig 3C). Young OASCs could generally reach over the 7^th^ passage, which was the maximum that cells from old donors could grow. Furthermore, OASCs in Group B displayed increased cellular senescent features as indicated by the higher expression levels of p21 and p53 mRNA, and more SA-β-gal positive staining cells (Fig 4). These findings suggested that pathways related to aging may have been activated in the OASCs derived from the prominent OF depots.

Next, age-related decrease in the OASC differentiation potentials towards adipogenic, osgteogenic and chondrogenic lineages was observed in the present study, suggesting that OASCs from elderly patients may be an inappropriate option for use to regenerate such tissues of interest. These results appeared to be inconsistent with previous studies which reported that donor age adversely affected the osteogenic and chondrogenic potentials of ASCs more than it did on the adipogenic differentiation [15–17,20]. We found that not only the percentage of oil red staining-positive cells but also the mRNA expression levels of PPARγ2, leptin and LPL, three key factors regulating adipogenic differentiation and maintaining the fat cell phenotype, were reduced with advancing age. The loss of adipogenic-differentiation capacity of OASCs could account for the decrease in lipogenic capacity of adipocytes, which would result in decreased fat cell size [29]. This was supported by the microscopic observation of OF tissue which demonstrated a declining trend in the fat cell size with age (Fig 2).

There are some limitations in this study. First, only female donors were enrolled to obtain the OF samples. The baggy-eye appearance is also very common in old male population, but fewer patients tend to seek aesthetic surgeries than females. Second, the amount of OF tissue harvested during eyelid blepharoplasty was limited. Low cell number upon isolation makes an *ex vivo* expansion step necessary to obtain therapeutic cell doses, which is expensive and time consuming, and enhances the risk of cell contamination and cell senescence [3]. Third, whether other linage differentiation capabilities of OASCs, such as the corneal epithelial, smooth muscle and neuronal lineages, were correlated with age still remained to be investigated in further studies. On the other hand, our previous studies proved that ASCs isolated from SF depots possess low immunogenicity and can maintain their biological functions after cryopreservation [32,33]. If these properties hold true in OASCs, allogenic or cryopresereved autologous OASCs may serve as alternatives for future clinical applications in old patients.

## Conclusion

In summary, our data showed that the regenerative potential of OASCs isolated from the protruded OF pads in old women is negatively influenced by donor age. The decreased progenitor cell number and increased cellular senescence may contribute to the prolonged proliferative time and reduced multilineage differentiation capabilities of OASCs. Alternative strategies such as cell banking or allogeneic cell sources may be more suitable when developing future OASC-based therapeutic approaches for the elderly patients.
